# The development of large-cell carcinoma in the wall of a giant bulla complicated by hemorrhage

**DOI:** 10.1186/s40792-016-0151-8

**Published:** 2016-03-10

**Authors:** Shota Nakamura, Koji Kawaguchi, Takayuki Fukui, Koichi Fukumoto, Toshiki Okasaka, Kohei Yokoi

**Affiliations:** Department of Thoracic Surgery, Nagoya University Graduate School of Medicine, 65 Tsurumai-cho, Showa-ku, Nagoya, 466-8550 Japan

**Keywords:** Large-cell carcinoma, Hemorrhage, Bullae

## Abstract

There were a few reports of patients with lung cancer developing at the wall of giant bullae complicated with hemorrhage. A 40-year-old male with complaints of hemoptysis was referred to our hospital, and a solitary pulmonary mass was pointed out on his chest roentgenogram. Computed tomography (CT) demonstrated a well-circumscribed solid mass measuring 7.0 × 6.5 × 6.0 cm in the right upper lobe of the lung. At the chest CT 1 year before, only a giant bulla without mass was found. From the interval change of CT findings with his clinical course, the mass was suspected as acute hemorrhage in the giant bulla. A right upper lobectomy of the lung was performed to control his hemoptysis. The surgical specimen showed the giant bulla filled with blood clot, and a partial wall of the bulla was irregularly thickened. Pathological examination revealed that the thickened wall was composed of large-cell carcinoma. In patients with bullous diseases complicated with hemorrhage, we should be aware of a possibility of developing lung cancer in the bullae.

## Background

The occurrence of lung cancers that arise within the walls of giant bullae is well known; however, the development is not usually accompanied with hemoptysis. We herein report a case of a patient whose lung cancer was found in a specimen after the resection of a giant bulla that was complicated by hemorrhage.

## Case presentation

A 40-year-old male who complained of hemoptysis of 1 month in duration was referred to our hospital, and a solitary pulmonary mass was observed on his chest roentgenogram. The vital signs were stable, but the breath sounds over his right upper lung field were diminished. Laboratory examination revealed a white blood cell count of 7500 cells/mL, a hemoglobin level of 8.8 g/L, a platelet count of 410,000 cells/mL. The patient’s electrolyte levels, liver test results, and coagulation study results were within the normal limits. The patient’s serum levels of tumor markers, including carcinoembryonic antigen, pro-gastrin-releasing peptide, and cytokeratin fragment, were also within the normal limits. There were no evidences of infection of acid-fast bacteria and fungus from his serum examinations and sputum cultures. The patient was a current smoker with a 20 pack-year history.

**Fig. 1:**
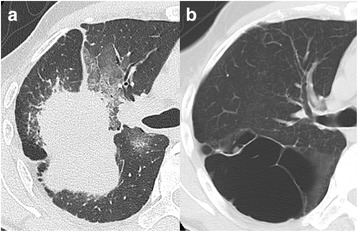
**a** A solitary mass in the right upper lobe of the lung. The mass was well-circumscribed and surrounded by a reticular shadow. The average HU value of the mass on HRCT was 35. **b** An emphysematous giant bulla of the lung was observed 1 year before the hemorrhagic presentation of the tumor

Computed tomography (CT) demonstrated a well-circumscribed mass measuring 7.0 × 6.5 × 6.0 cm accompanying a reticular shadow in the right upper lobe, with multiple emphysematous bullae in the bilateral upper sides of the lung (Fig. 1a). Chest magnetic resonance imaging revealed a mass with heterogeneous attenuation. One year previously, the patient had undergone CT after complaining of shortness of breath (Fig. 1b). The previous CT showed a giant bulla without any wall thickening in the right upper lobe. Based on a comparison of the image findings and his clinical history, an acute hemorrhage was suspected to have occurred inside the bulla; there were no CT findings that were suggestive of a malignant tumor. The initial treatment was percutaneous trans-catheter embolization of the feeding bronchial artery of the mass. Surgery was planned after the initial treatment failed to control his hemoptysis.

**Fig. 2:**
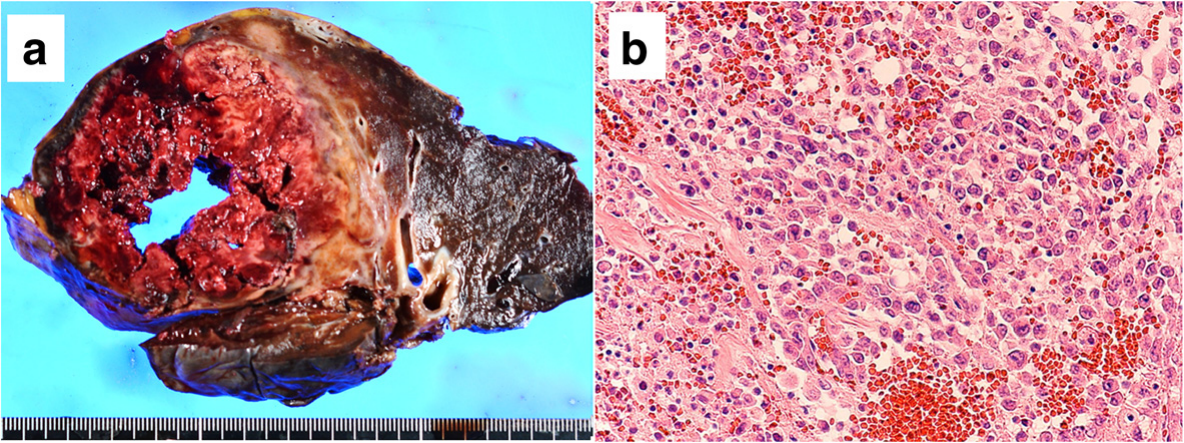
**a** A gross image of the resected specimen showing the right upper lobe with a giant bulla filled with clotted blood. **b** A microscopic image of the resected specimen shows poorly differentiated tumor cells with poor epithelial connection. The tumor was composed of large cells with coarsely clumped nuclei and prominent nucleoli

A right upper lobectomy was performed via a posterolateral thoracotomy, which resulted in the complete resection of the mass. Because the mass was found to have severely adhered to the chest wall, the parietal pleura was also resected. The operation time was 344 min with 2001 ml of blood loss. The resected specimen showed a giant bulla filled with clotted blood; irregular thickening was observed in part of the wall (Fig. 2a → A).

A pathological examination of the mass revealed a giant bulla filled with clotted blood accompanied with inflammatory cell infiltration and fibroblast proliferation. A specimen from the thickened wall was subjected to morphological and immunohistochemical investigations, which led to the diagnosis of large-cell carcinoma (Fig. 2b). Results of the immunohistochemical examinations were as below: EMA, CAM5.2, and AE1/AE3 were positive. CK5/6 and TTF-1 were negative. The tumor was 5.5 × 4.5 × 1.5 cm in size. Although invasion to the parietal pleura was observed, there was no vessel or lymphatic invasion. Postoperative brain MRI and positron emission tomography scans revealed no evidence of distant metastasis. The tumor stage was thus proven to be pT3N0M0 stage IIB.

The postoperative course was uneventful, and the patient’s hemoptysis resolved. The patient received four courses of postoperative adjuvant chemotherapy with cisplatin and vinorelbine. He is currently alive without recurrence 24 months after the operation.

### Discussion

There are only 11 reported cases of hemorrhage in bullae (of any cause) [1–10]. Although the frequency of those cases is not clear, it seems to be quite rare because of the small number of reported cases. In four of the 11 cases, the suspected cause was hemorrhagic tendency due to anticoagulation therapy or acquired hemophilia [1–4]. The other six cases were complicated by pulmonary malignancies and did not involve hemorrhagic tendency [6–10]. These facts suggest that we should be aware of the possible development of lung cancer in the blood-filled bullae of patients with bullous diseases complicated by hemorrhage.

Five of six lung cancer patients underwent surgery to control their hemoptysis within 1 month of visiting their respective hospitals. Lung cancer was not initially suspected due to the complex CT images, but malignancies were eventually diagnosed after the examination of the resected specimens. The collection of fluid within the bullae made it difficult to determine the thickness of the wall or the mass along the wall.

The relative risk of lung cancer development in the wall of the bullous lung is reported to be much higher in patients with bullous disease. The incidence of lung cancer in patients with giant bullae is reported to be 7–29 % [11]. In addition, Hanaoka et al. reported that lung cancer patients with pulmonary bullous disease have a poor prognosis because their disease is advanced at the time of detection and because the tumor has aggressive characteristics [11]. Indeed, the tumor cells of the present case were poorly differentiated, and the histological type was large-cell carcinoma. Similarly, almost all of the other cases involved poorly differentiated lung cancers. Their diagnoses included large-cell carcinoma (*n* = 3), pleomorphic carcinoma (*n* = 1), and adenocarcinoma (*n* = 1).

## Conclusions

In conclusion, the present case suggests that we should consider the possibility of a pulmonary malignancy when a giant fluid-filled bulla is observed on imaging examinations.

## Consent

Written informed consent was obtained from the patient for publication of this case report and any accompanying images. A copy of the written consent is available for review by the Editor-in-Chief of this journal.

## References

[1] Thomas DW, Balikai G, Nokes TJ (2008). Bleeding into an emphysematous bulla. Brit J Haematol.

[2] Trzcinski M, Folcik K, Burakowska B (2012). The bleeding into the emphysematosus bulla imitating lung tumor. Pneumonolgia I alergologia polska.

[3] Kaira K, Takei Y, Matsuura M, Saito R (2003). Pulmonary hematoma resulting from anticoagulant therapy. Am J Rentogenol.

[4] Nakajima J, Yamamoto M, Kotsuka Y (1997). Hemoptysis from an emphysematous bulla developing after open-heart surgery: report of a case. Surg Today.

[5] Nagashima O, Suzuki Y, Iwase A, Takahashi K (2012). Acute hemorrhage in a giant bulla. Intern Med.

[6] Nakamura H, Takamori S, Miwa K (2003). Rapid-growth lung cancer associated with a pulmonary giant bulla: a case report. Kurume Med J.

[7] Kanzaki M, Onuki T, Shioiri M (1996). A case report of undifferentiated lung cancer difficult in diagnosis by hemoptysis. Jpn J Chest Surg.

[8] Sakamaki F, Nakano M, Urano T (1995). A case of large cell carcinoma of the lung arising from the inner surface of a pulmonary bulla and complicated by hematoma. Jpn J Thorac Dis.

[9] Shindo G, Endo T, Onda M (2005). Pulmonary large cell carcinoma contiguous to bullae with massive bullous hematoma and hemoptysis. Kyobu Geka.

[10] Watanabe S, Satoh H (1997). A case of early pulmonary large cell carcinoma found in a giant bulla containing coagulations and disseminated cancer. Jpn J Chest Surg.

[11] Hanaoka N, Tanaka F, Otake Y (2002). Primary lung carcinoma arising from emphysematous bullae. Lung Cancer.

